# MicroRNAs and Their Regulatory Role in Sugarcane

**DOI:** 10.3389/fpls.2017.00997

**Published:** 2017-06-13

**Authors:** M. Swapna, Sanjeev Kumar

**Affiliations:** Division of Crop Improvement, Indian Institute of Sugarcane Research, Indian Council of Agricultural Research (ICAR)Lucknow, India

**Keywords:** biomass, disease resistance, microRNAs, metabolic pathways, regulation, stress tolerance, sugar content, sugarcane

## Abstract

Sugarcane, one of the most photosynthetically efficient crops, is an important source of sugar and feedstock for green energy and co-generation. The high level of polyploidy and genomic peculiarities in this crop point towards a complex mechanism of regulation for the economically important traits like sugar content, cane yield related traits, resistance to biotic and abiotic stresses etc. The regulatory pathways for these traits comprise of a number of genes, transcription factors and different categories of RNAs like small interference RNAs (siRNAs), and Micro RNAs (miRNAs). MicroRNAs (miRNAs) are found to play an important regulatory role in many crops. As in other crops, several miRNAs have been identified in sugarcane too and these are speculated to have a role in regulating the various metabolic processes. Role of miRNAs in relation to drought tolerance has been studied to a great extent in this crop. miRNAs have been predicted to be linked to expression of other traits like disease resistance, salinity tolerance, waterlogging and axillary bud growth in sugarcane. miRNAs can have a significant role in biomass production in sugarcane, as reported in several biofuel crops. Till now, miRNAs linked to sugar accumulation have not been identified in sugarcane, but studies suggest an important role for miRNAs in sugar metabolic pathway in crops like *Sorghum* and switch grass. It is presumed that in sugarcane too, sugar accumulation as well as the other important metabolic pathways might be regulated to some extent by the miRNAs. The review examines the progress made in understanding the miRNA regulation in sugarcane and the extent to which miRNA mediated regulation can be utilized in sugarcane improvement.

## Introduction

Sugarcane is one among the most photosynthetically efficient crops contributing to the major share of sugar production in the world. The plant is unique that the storage of sugar in the parenchymatous tissues takes place at a very high concentration. The high biomass content makes it a fitting source of feedstock for green renewable energy. Sugarcane produces the highest crop tonnage (FAOSTAT, 2008), on an average, 40 t/ha of dry stalk and trash (Waclawovsky et al., 2010), with an estimated capacity to store sugar upto 62% of dry weight or 25% of fresh weight of the stalk. The improvement efforts till now have mainly focussed on boosting the sugar content in the crop. There is considerable gap between the theoretical conversion efficiency and the actual solar energy conversion reported in sugarcane. With a wide range being observed for sucrose content on dry matter basis (350–400 mg/g to upto 500–560 mg/g sucrose), ample scope exists in this crop for increasing the photosynthetic efficiency as well as the sucrose accumulation potential through conventional as well as modern crop improvement tools.

The global concentration of CO_2_ has reached 405.07 ppm in February 2017 (NAAO, 2017). As the atmospheric CO_2_ levels approach the 700 ppm mark, the advantage which the C_4_ plants enjoy over the C_3_ plants with respect to increased photosynthetic efficiency may start disappearing (Zhu et al., 2008). This necessitates manipulations to facilitate the C_4_ plants to better utilize the solar energy and for better partitioning of the biomass into desirable components, even with increasing CO_2_ levels. With the necessity to produce more, the crop may be grown in less productive lands too, under various abiotic and biotic stresses, in future. Regulatory mechanisms need to be elucidated for necessary interventions at key points. Thus, not only sugar accumulation, but traits like biomass production, nutrient and water use efficiency, stress tolerance, ratoon productivity and post harvest management also need to be fine-tuned for increased productivity, with maximum utilization of resources. Hence, an understanding of the factors regulating various metabolic pathways and their manipulation at the key steps assumes great significance.

Many genes and regulatory factors including different categories of RNAs are involved in these metabolic processes. Interaction of the RNA sequences with the target sequences may regulate the various processes through post-transcriptional as well as post-translational modifications. MicroRNAs (miRNAs) are predicted to be one among these regulatory factors, associated with various economically important traits in sugarcane (Ferreira et al., 2012; Thiebaut et al., 2012; Gentile et al., 2013, 2015; Srivastava, 2013; Lin et al., 2014).

## MicroRNAs (miRNAs): Origin and Mechanism of Gene Regulation

Small RNAs (sRNAs) are 20–30 nucleotides long RNA sequences, which have important roles in the various regulatory pathways. The group consists of microRNAs (miRNAs, 21–22 nucleotides), small interference RNAs (siRNAs, 21–24 nucleotides) and Piwi-interacting RNAs (piRNAs, 28–30 nucleotides) (Axtell and Bowman, 2008). These are known to regulate the expression of a number of key developmental and stress related genes in many crops (Jones-Rhoades et al., 2006).

**FIGURE 1:**
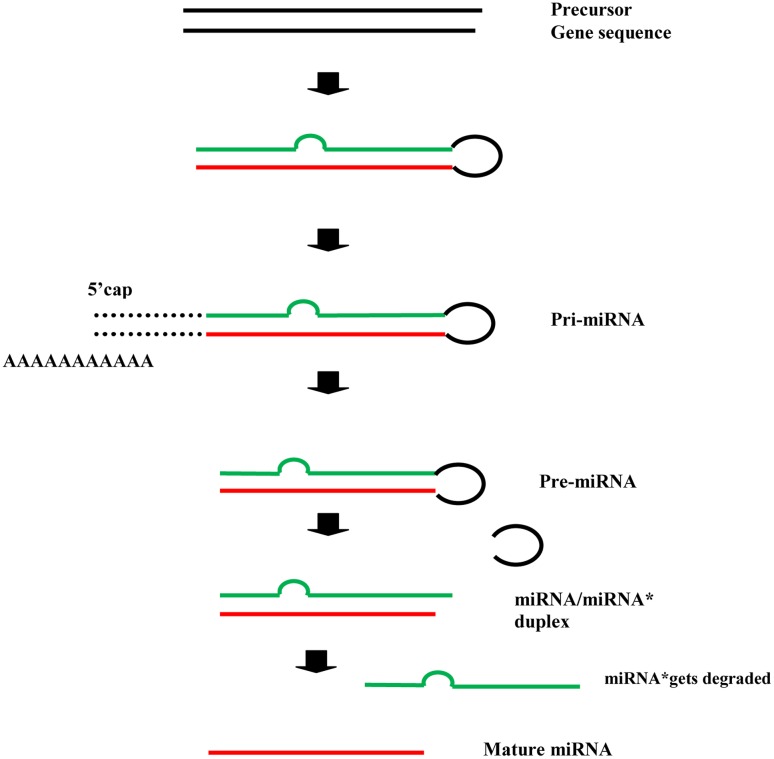
MicroRNA formation: a schematic illustration.

In plants, the precursor gene sequences, after a series of intermediaries, give rise to the mature microRNAs i.e., miRNAs (the leading strand), whereas the miRNA^∗^ (the passenger strand) gets degraded (Ferreira et al., 2012; Saikumar and Kumar, 2014). The process can be roughly illustrated as follows (**Figure 1**). The mature miRNA binds with ARGONAUTE (AGO) protein and directs the cleavage or translational repression of target mRNAs.

The nucleotide at the 5′ end of the miRNA is significant as it determines the AGO protein to which, the miRNA binds (Mi et al., 2008). In many crops, the most frequently present nucleotide at the 5′ end is the Uracil (U), whereas in sugarcane, both Uracil (U) and Adenine (A) have been observed to be present in almost equal frequency (Ferreira et al., 2012). In sugarcane, there seems to be a preference among 21 nt new miRNA candidates for U at their 5′ end and among 24 nt candidates for A in the same position (Thiebaut et al., 2012). In rice and *Arabidopsis*, AGO1 associates preferentially with an miRNA with U at the 5′ end, while AGO2 binds preferentially with an miRNA, with A at the corresponding position. AGO5 has been found to prefer cytosine as 5′ cap. Exceptions have also been reported as in *Arabidopsis*, where miR172 (5′cytosine) gets associated with AGO1 in most of the cases and majority of miR390 (5′ adenosine) bind with AGO7 (Mallory and Bouche, 2008). Besides, the presence of conserved histidine residues at specific positions in the AGOs has been detected, which is critical for slicing of miRNA:AGO complex (Kapoor et al., 2008; Thiebaut et al., 2012). Such preferences and conserved sequences may exist in sugarcane too and these may influence the regulatory mechanisms in this crop.

The regulation of gene expression by miRNAs in plants is mainly through target cleavage and transcriptional and translational repressions (Chen, 2004; H-shan et al., 2005). Additionally, miRNAs may regulate gene expression through DNA methylation (Wu et al., 2010). In *Arabidopsis*, target mimicry has been found to operate, as in the regulation of shoot pi content through miR399 (Franco-Zorilla et al., 2007). An additional regulatory pathway was identified in rice and *Arabidopsis* where, miRNA recognition sites in the introns of mRNAs are targeted by specific miRNAs (Meng et al., 2013). These introns get cleaved depending on the presence or absence of cleavage sites. Single nucleotide polymorphisms (SNPs) residing in the target sites and other mutations in the miRNA binding sites of the target gene can lead to loss or gain of miRNA target sites, thereby modifying the regulatory process (Pelletier and Weidhaas, 2010). Regulation by miRNAs may not always be through single target regulation. miRNA–miRNA interactions, where the expression of one miRNA is regulated by another miRNA, has been reported (Guo et al., 2012; Xu et al., 2014). Trans-acting miRNAs (*ta-siRNAs*) from *TAS* genes direct the cleavage of partially complimentary mRNAs as a regulatory mechanism (Vaucheret, 2005; Mallory and Bouche, 2008; Montgomery et al., 2008). Thus, the mechanisms may vary depending upon the crop and the gene regulated. The same metabolic pathway may have different genes/factors on which different miRNA regulatory mechanisms operate.

## miRNAs in Sugarcane: Regulating the Various Pathways

miRNAs and their role in gene regulation have been studied in many crops (Jones-Rhoades et al., 2006; Axtell and Bowman, 2008; Mallory and Bouche, 2008; Ding et al., 2013; Saikumar and Kumar, 2014; Srivastava et al., 2015). In sugarcane also, miRNAs have been speculated to have a major role in regulating various traits (Zanca et al., 2010). The identification of mature miRNAs and their expression analysis has been limited in this crop. The absence of a well sequenced complete genome of sugarcane, along with the inherent complexities of the crop makes the miRNA studies in sugarcane more complicated. The highly unstable nature of the miRNA precursors makes their identification in the EST collections difficult (Gentile et al., 2015). Discovery of novel miRNAs and prediction of targets for miRNA, necessitate the availability of a sequenced genome, even though, wet bench cloning and *in silico* sequence mining can be an answer to this challenge to some extent, at the initial stages. Application of comparative genomics has facilitated the use of information from *Sorghum*, the closest diploid relative of sugarcane, for miRNA identification and further analyses in sugarcane (Zanca et al., 2010). Some limited studies on miRNA based regulation in sugarcane have revealed information regarding the categories of miRNAs present and their abundance in relation to particular traits. More of 24 nt long miRNAs were observed in salt stress and drought stress sensitive libraries from sugarcane genotypes. Drought stress tolerant libraries had more of 21 nt miRNAs. Thus, the extent of involvement of these two groups of miRNAs in regulating a particular pathway depends, on the trait analyzed and on the target identified. The putative miRNA target MADS2, a MADS-based transcription factor that regulated development, was recognized by 21 nt miRNAs whereas, 24 nt miRNA candidates were found to recognize a 60S acidic ribosomal protein RPP2B involved in biotic stress resistance (Thiebaut et al., 2012).

### miRNAs and Drought Response in Sugarcane

Perhaps the most widely studied miRNA mediated regulation in sugarcane is the one related to drought (Ferreira et al., 2012; Thiebaut et al., 2012; Gentile et al., 2013, 2015; Srivastava, 2013; Lin et al., 2014). Computational studies using the sequences from four each of drought tolerant and drought sensitive sugarcane genotypes that had been subjected to biotic and abiotic stresses, identified 623 candidates of new mature miRNAs (Thiebaut et al., 2012). Of these, 44 were classified as high confidence miRNAs. 67 miRNAs were identified specifically in the water deficit assay, with 20 being shared by the tolerant and susceptible assays. The putative targets and their biological functions were identified and these were predicted to be targeting serine/threonine kinases, zinc-finger proteins etc. Ferreira et al. (2012) identified 18 miRNA families in sugarcane cultivars differing in their level of drought tolerance, with seven of these differentially expressed during drought. The differential expression varied with the extent/duration of water stress. Study of expression profile of miRNAs in a drought resistant cultivar ROC22 by Lin et al. (2014) identified 23 conserved and 34 new miRNAs in the leaves, with 438 putative target genes. Eleven miRNAs were found to be differentially expressed among the control plants and the plants subjected to drought. Expression studies of the micro-transcriptome regulating drought response by Gentile et al. (2013), using two sugarcane cultivars of contrasting drought tolerance, identified 18 miRNA families with 30 mature miRNA sequences, 13 of them being differentially expressed. Seven miRNAs were differentially expressed in both the sugarcane cultivars. Two of these were observed under all the stress durations and all growing conditions. Transcription factors, transporters and proteins associated with senescence and flower development. were reported to be among the target sequences for these miRNAs, speculating the existence of cross talk among these pathways. Some of the miRNAs were up-regulated in one cultivar while the same were down-regulated in another cultivar under drought stress. The duration of the stress and the growing conditions (greenhouse vs. field) also influenced the miRNAs expressed, with a shift in the up or down regulation of some miRNAs as per the duration of stress. Short term PEG stress led to significant up-regulation of miR159 targeting the *MYB* transcription factor family, in sugarcane leaves (Patade and Suprasanna, 2010). Thus, the miRNA regulation seems to be dependant largely on the type and duration of stress induced. Tolerance/susceptibility of sugarcane plants to waterlogging has also been speculated to be associated with miRNA regulation in a study, wherein, seven candidate miRNAs were identified in plants subjected to waterlogging for one month. These were speculated to be sugarcane specific (Khan et al., 2014).

All these studies point towards a possible role of miRNAs, both induced and modulated by drought, in regulating the drought response in this crop. These may be one among the many factors involved in the regulatory cascade of the entire pathway. These investigations helped in identifying some of the target genes for these regulatory sequences, with genes for plant growth and related processes also being predicted as probable targets. These also brought out the cross-talk that might be present among the various metabolic pathways.

### miRNAs Regulating Other Stresses in Sugarcane

Other than drought response, salinity and response to diseases have also been studied with reference to miRNA mediated regulation in sugarcane. Short term salinity stress has been found to result in up-regulation of transcript expression of *MYB*-related genes, with a concomitant down regulation of miRNA (Patade and Suprasanna, 2010). Carnavale-Bottino et al. (2013), in their studies in *Saccharum* spp. cultivars grown in mild and intense salt stress, identified 11 miRNAs with higher expression in severe salt stressed plants, compared to that in mild stress.

The gene sequences in sugarcane that act as targets for pathogen miRNAs and also the miRNAs in sugarcane related to diseases incidence have been investigated. Thiebaut et al. (2012) identified more than 240 miRNAs in sugarcane infected with *Acidovorex avenae ssp avenae*. Viswanathan et al. (2014) computationally predicted and experimentally validated the miRNAs encoded by the Sugarcane Streak Mosaic Virus (SCSMV) infecting sugarcane genome and identified their potential gene targets in sugarcane. A total of 30 putative miRNAs were identified in the pathogen. 19 target genes belonging to several gene families were identified for the miRNA SCSMV miR16 in sugarcane. Some of the miRNA families are found to target the genes for NBS-LRR plant immune receptors in legumes (Zhai et al., 2011; Shivaprasad et al., 2012) and solanaceous plants (Li et al., 2012) with a possibility of similar occurrence in sugarcane also.

### miRNAs and Regulation of Traits Related to Biomass

With the crop emerging as an efficient feed stock for cellulosic bioethanol, studies on the metabolic pathways related to lignin and degradation of cellulosic material have gained importance in sugarcane. miRNAs for recalcitrance and biconfinement, along with those for development and stress response were identified in some of the biofuel crops (Trumbo et al., 2015). miR156 and miR159 have been found to be associated with recalcitrance, bioconfinement and also abiotic stresses in sugarcane. Several other miRNAs linked to biomass improvement *viz*., miR164, miR166, miR167, miR172, miR398, miR414, miR444, miR477, miR528, miR531, miR854, miR1535, miR1848, miR102, miR2118 etc., have been identified in related biofuel crops by these researchers and these may have important roles in sugarcane also. Overexpression of miR156 has been found to have a 30% reduction in lignin content in switch grass. It has been speculated that the relative levels of miR156 and miR172 regulates juvenile-to-adult transition and thereby, the biomass content. miR156 is reported to down regulate Trehalose-6-Phosphate Synthase-1, which is also involved in sucrose metabolism. miR166, miR169, and miR139, are also reported to regulate shoot development, flower development, phase transition and sugar production. Thus, all these miRNAs are reported to favor biomass accumulation through different strategies (Trumbo et al., 2015). Twenty six conserved miRNA families and two putative miRNAs were identified by Ortiz-Morea et al. (2013), in their studies of small RNAs in active and developing vegetative buds in sugarcane. The expression pattern suggested a role for the miRNAs in regulating abscicic acid signaling pathway during bud growth. A fine-tuned regulation of miR139, resulting in a molecular switch in the inactive vegetative buds and a higher expression of the gene *SsGAMYB* in actively growing axillary buds was suggested in this study. The regulation of axillary bud development may have a role in the vegetative growth of the plant thereby, possibly influencing biomass accumulation.

### miRNAs and Sugar Metabolic Pathway in Sugarcane

Even though sugar accumulation and final sugar content are important economic traits in sugarcane, till now, no miRNAs have been reported to be related to sugar metabolism in this crop. In switch grass (*Panicum virgatum* L.), miR156 and miR139 are reported to be associated with regulation of the enzymes sucrose synthase and trehalose -6-Phosphate (Xie et al., 2014). Calvino et al. (2011) and Yu et al. (2015) identified miRNAs differentially expressed in grain and sweet sorghum (e.g., miR169), speculating that this miRNA might be associated with sugar accumulation in this crop. Three miR169 genes – miR169a, miR169b, and miR169c (out of a total of 18 detected), have been found to be differentially expressed in the stems and leaves of sweet and grain sorghum. These reports also ascertain the importance of source (leaves) as well as sink (stems) in the sugar accumulation pathway. Also, the relative abundance of 24 nt miRNAs that influence transcriptional gene silencing, compared to the 21 nt miRNAs which are responsible for post-transcriptional gene silencing in sweet sorghum tissues may be an indicator of the regulatory mechanisms more prevalent in this crop. The studies by Calvino and his group (Calvino et al., 2011) reported an equal abundance of miR395 as well as miR395^∗^ in sweet sorghum stem tissues, suggesting a regulatory role for the miR395^∗^ strand also in sugar accumulation. Sorghum is the closest diploid relative of sugarcane and comparative genomic studies have reported similarities with respect to gene sequences and their function in the two crops. Thus, it is possible that these miRNAs identified in sweet and grain sorghum may have regulatory roles in the sugar accumulation in sugarcane also. Differential accumulation of potential target genes seldom has a simple correlation with miRNA levels, as reported in sweet sorghum (Calvino et al., 2011). Such findings call for more detailed studies and some amount of caution, before directly applying the results from other related crops to sugarcane.

## miRNA Regulation in Sugarcane: Possibilities and Challenges

There are indications that miRNAs play a role in regulating temporal transitions, especially with respect to control of developmental timings, in many crops. miR172 regulates flowering time by targeting a sub-family of APETALA2 (AP2) transcription factor genes in *Arabidopsis* (Aukerman and Sakai, 2003). Similar type of miRNA regulation along with other mechanisms, may occur in sugarcane also, where temporal differences are exhibited with respect to the maturity of sugarcane genotypes. There can be differential regulatory mechanisms by some miRNAs in early and late maturing varieties in which, the sucrose content reaches the peak level at 8–10 months after planting or at 10–12 months after planting, respectively. Varietal differences may exist with respect to the extent of inversion of the sugar accumulated in this crop and this also might be regulated by miRNA, along with many other factors. It is possible that the relative levels of different miRNAs and fine-tuning of their expression regulate the sugar accumulation, and thereby, the time of maturity of the crop. These speculations need to be tested and validated so that the miRNA regulatory mechanism can be fully exploited to manipulate major economically important traits in this crop.

The different categories of genes speculated to be targeted by the individual miRNAs, point towards the complexity of the entire regulatory mechanism and emphasize the existence of “cross talk” among the various pathways. Still a lot need to be understood to pinpoint the miRNAs regulating the various traits and those differentially expressed.

The correlation and differential expression of the miRNAs in these studies with respect to the different traits, give a preliminary insight into the presence of miRNAs and their possible role in regulating the various metabolic processes. These do not present a conclusive evidence that the miRNAs do regulate the traits studied in sugarcane, and more studies may be needed in this regard. It can be inferred that miRNAs are not solely responsible for regulating the metabolic pathways and these may be one among the several components of the entire regulatory network (Ferreira et al., 2012). Also, as reported by Calvino et al. (2011), a lack of simple correlation between the miRNA levels and the target genes points towards a complex process of regulatory mechanism involving miRNAs. There can be varying mechanisms of regulation involving different miRNAs, resulting in transcriptional or post-transcriptional regulation. The variation exhibited with respect to the miRNA expression profile, with changes in the growth conditions and trait of interest, indicates that the process of regulation by miRNAs is highly complicated. Several points are yet to be answered in relation to miRNAs and gene regulation in sugarcane. It has been reported that duplicate genes are more likely to be targeted by miRNAs than singletons (Wang and Adams, 2015). With the high ploidy level of sugarcane, will the possibility of miRNA regulation for traits be more in sugarcane than that in other diploid crops? Are all the metabolic pathways and the traits/genes for a particular trait regulated by miRNAs? Do all the genes governing a particular trait have miRNA binding sites? Is there a basis for preference of specific traits/genes for being regulated by miRNAs or is it random? How do the ploidy level and the complexity of genome affect the presence of miRNAs and their regulatory role? To what extent can the information from comparative genomics with respect to regulatory role of miRNAs be applied to sugarcane improvement? These are some of the points that need to be considered in detail.

## Conclusion

With the modern tools for computational prediction and expression studies, and advances in bioinformatics, researchers can take full advantage of miRNA regulation in the various economically important traits. Still, challenges do exist in this area. Variations in sequencing methodology, interactions between miRNAs and their transcripts, pleiotropy, multiple-miRNA alterations etc., are a few of the challenges that need to be addressed. These will be in addition to the complexities for a crop like sugarcane due to its inherent genomic peculiarities. The advances in the tools available and the information from the genome sequencing projects in this and other related crops, along with comparative genomics, will help in overcoming these challenges, so that the regulatory properties of miRNA can be effectively exploited for improvement of important traits in sugarcane too.

## Author Contributions

SM has conceptualized the idea, consulted the literature and prepared the manuscript. SK has assisted in consulting literature and preparing the manuscript.

## Conflict of Interest Statement

The authors declare that the research was conducted in the absence of any commercial or financial relationships that could be construed as a potential conflict of interest.
